# Structural homology guided alignment of cysteine rich proteins

**DOI:** 10.1186/s40064-015-1609-z

**Published:** 2016-01-12

**Authors:** Thomas M. A. Shafee, Andrew J. Robinson, Nicole van der Weerden, Marilyn A. Anderson

**Affiliations:** Department of Biochemistry and Genetics, La Trobe Institute for Molecular Sciences, La Trobe University, Melbourne, 3086 Australia; College of Science, Health and Engineering, La Trobe University, Melbourne, 3086 Australia; Life Sciences Computation Centre, Victorian Life Sciences Computation Initiative, Melbourne, 3053 Australia

**Keywords:** Alignment, Barcode, Cysteine-rich proteins, Defensin

## Abstract

**Background:**

Cysteine rich protein families are notoriously difficult to align due to low sequence identity and frequent insertions and deletions.

**Results:**

Here we present an alignment method that ensures homologous cysteines align by assigning a unique 10 amino acid barcode to those identified as structurally homologous by the DALI webserver. The free inter-cysteine regions of the barcoded sequences can then be aligned using any standard algorithm. Finally the barcodes are replaced with the original columns to yield an alignment which requires the minimum of manual refinement.

**Conclusions:**

Using structural homology information to constrain sequence alignments allows the alignment of highly divergent, repetitive sequences that are poorly dealt with by existing algorithms. Tools are provided to perform this method online using the CysBar web-tool (http://CysBar.science.latrobe.edu.au) and offline (python script available from http://github.com/ts404/CysBar).

**Electronic supplementary material:**

The online version of this article (doi:10.1186/s40064-015-1609-z) contains supplementary material, which is available to authorized users.

## Background

Cysteine Rich Proteins (CRPs) are found in all kingdoms of life and are involved in diverse functions—from innate immunity to signalling to neurotoxicity (Craik et al. 2001; Koppers et al. 2011; Van der Weerden and Anderson 2013). Their properties are markedly different from globular proteins since their stability stems from the covalent disulphide bonds that cross-link their sequence, rather than relying on a hydrophobic core (Colgrave and Craik 2004; Fass 2012). This robustness allows families to evolve high sequence diversity in the inter-cysteine loops as well as making them interesting scaffolds for protein engineering (Gracy and Chiche 2011; Northfield et al. 2014). In this work we use the example of defensins, a family of small, cationic CRPs found in plants in invertebrates which perform key roles in defence against pathogens.

Protein sequence alignment is the first step in many bioinformatic analyses necessary to understand sequence-function relationships. Errors in alignment may lead to erroneous conclusions being drawn and so having accurate alignments is very important. As well as increasingly accurate multiple sequence alignment tools being developed, some software tools take existing alignments and refine them. For example the RASCAL and Refiner software (Thompson et al. 2003; Chakrabarti et al. 2006), however CRPs still pose problems for most alignment algorithms, leading to a high reliance on extensive manual alignment (Dassanayake et al. 2007; Whittington et al. 2008). This is due to two factors. Firstly, the only conserved part of the sequences is often the cysteines comprising the disulphide bridges and sequence identity within a family is frequently below 15 % (Van der Weerden and Anderson 2013). Secondly, inter-cysteine loops typically have a high rate of insertion and deletion leading to large gaps in the alignment. Together these factors lead to misalignment of sequences such that alignment columns frequently contain structurally non-homologous cysteines (Russell and Ponting 1998; Liu et al. 2009; Dwivedi and Gadagkar 2009).

**Fig. 1:**
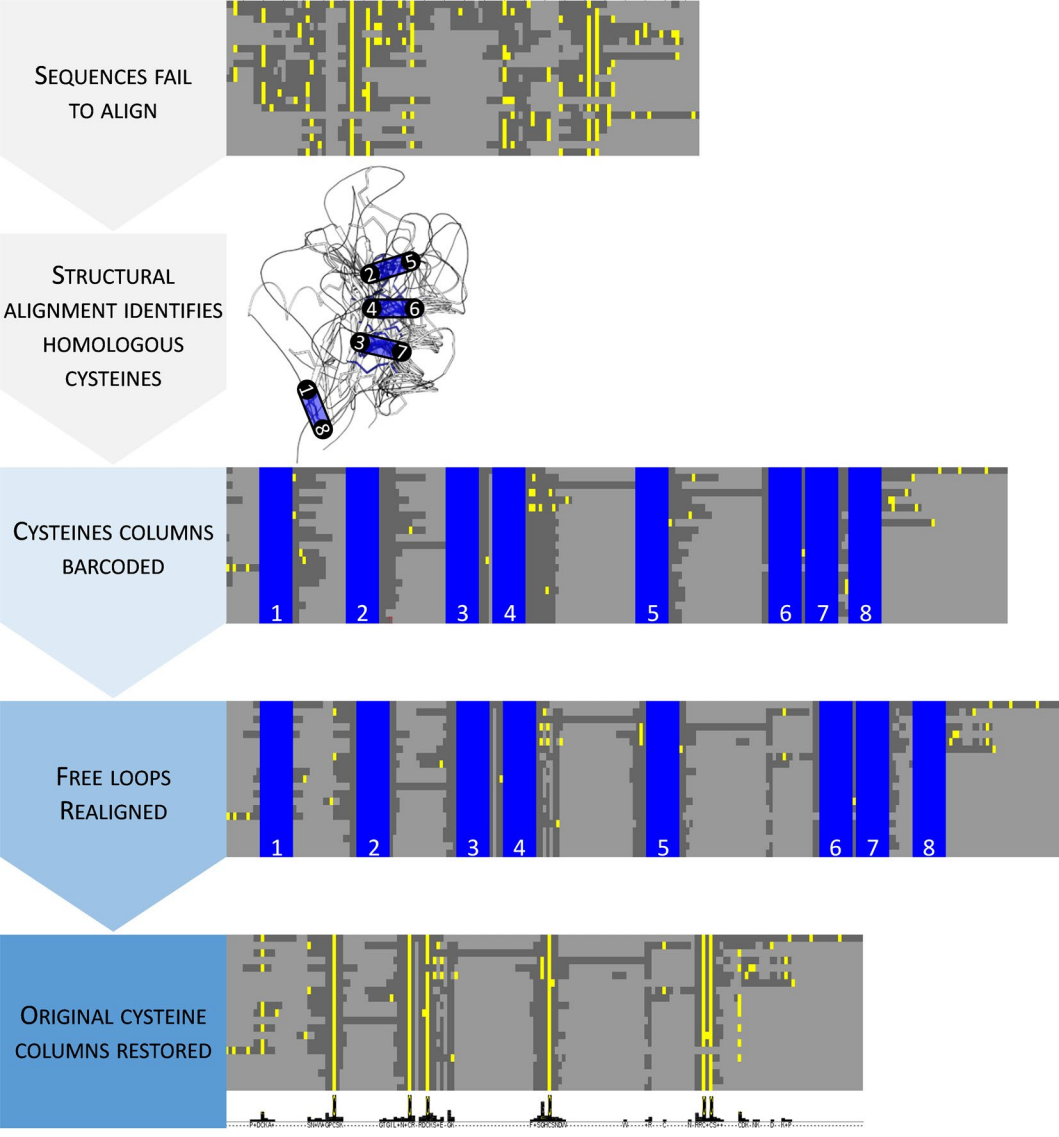
Overview of barcode alignment method. Lack of sequence conservation and abundance of cysteines prevents automatic alignment by standard methods. Homologous cysteines identified from structural alignment are replaced with 10aa barcodes to pin them in place. Standard algorithms are used to realign free loops between the *barcoded columns*. *Barcodes* are exchanged for the original columns for the final alignment and phylogeny calculation. Sequences are *coloured* with cysteines in *yellow*, any other residue in *grey*, gaps in *light grey*, and barcode sequences in *blue*

Here we solve these problems with a method for aligning divergent, disulphide-rich protein sequences by identifying structurally homologous cysteines using the DALI server (Holm and Rosenström 2010) and replacing them with 10 amino acid barcodes (Fig. 1). Tertiary structure is widely considered a good indicator of homology since structure is more conserved than protein or DNA sequence (Grishin 2001; Hasegawa and Holm 2009). Barcoding homologous cysteines by CysBar links the known structural information to the sequences.

The barcoded sequences can then be aligned by existing algorithms with the barcoded columns effectively forced to align. Finally, the barcode sequences are removed from the alignment and the original columns restored by CysBar to yield a final alignment. This process reduces the need for manual manipulation leading to a more impartial alignment. Barcoding and reconstruction steps can be performed online with the new CysBar web-tool (http://CysBar.science.latrobe.edu.au) or with the offline python script (*cysbar.py*) and results analysed with the *loopproperties.xlsx* spreadsheet.

## Implementation and results

### Repeat cysteines and divergent loop sequences cause cysteine misalignment

CRP superfamilies are typically highly sequence diverse, possibly due to the stability of the small, disulphide-constrained fold. Indeed, the cysteines are often the only conserved residue. In the absence of similar sequence, alignment algorithms are typically heavily biased by attempting to align cysteines. Additionally, the high insertion and deletion rate and variations in disulphide pattern causes frequent cysteine misalignment (Additional file 1: Figure S1). Misalignment of 15–25 % of the cysteines (Additional file 1: Figure S2a) causes further misalignment between non-homologous inter-cysteine residues. For example, the frequent insertions and deletions within known secondary structure (Additional file 1: Figure S2b) conflicts with known trends in structure evolution (Pascarella and Argos 1992; Zhang et al. 2012).

### Structure used to align homologous cysteines

**Fig. 2:**
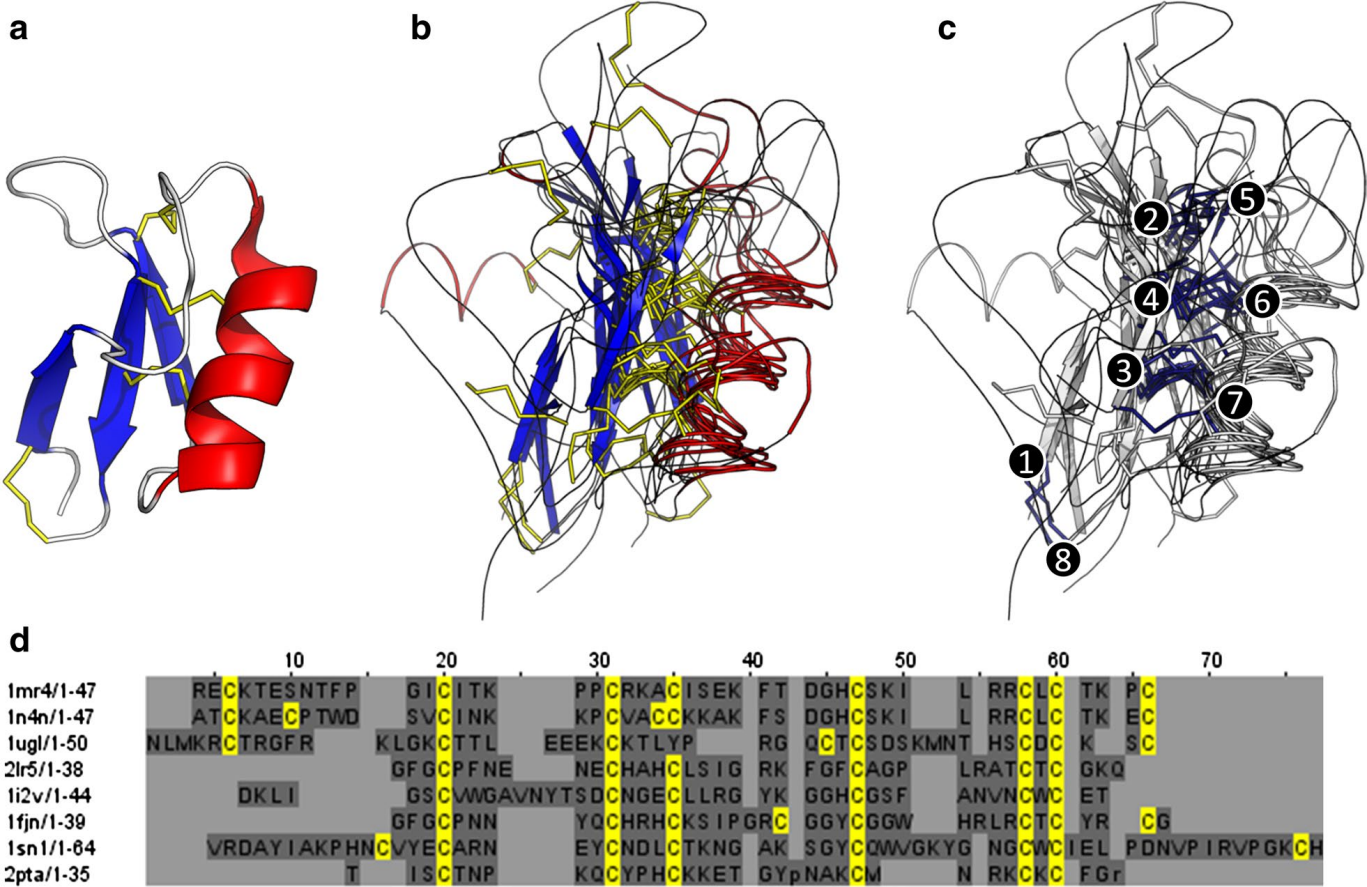
Identifying homologous cysteines by structural alignment. **a** The starting query structure (1MR4). **b** Overlay of aligned structures identified by DALI (Holm and Rosenström 2010). **c** The cysteine pairs indicated by DALI to be homologous in the structures. **d** Alignment of sequence based on structure by DALI. *PDB accession numbers: 1MR4, 1N4N, 1UGL, 2LR5, 1I2V, 1FJN, 1SN1, 2PTA*

To address the deficiencies of standard alignment protocols, it is necessary to use structural homology to identify the homologous cysteines that should be barcoded. The DALI (Holm and Rosenström 2010) structural homology webserver can be queried using a PDB structure and return both a list of homologous structures, and a sequence alignment based on backbone positions of the overlaid structures (Fig. 2). This alignment is used to identify homologous cysteines. In the sample dataset of defensins, cysteines are counted as homologous if they are either in the same column, or a neighbouring column (having shifted <3Å along the structure). Checking which columns contain a set of homologous cysteines is the only step that requires manual decision making.

Sequences without known structure are assigned to the closest relative with known structure by querying BLAST (Altschul et al. 1990) with the sequences of known structure. This process generates sub-group alignments containing at least one sequence of known structure (Additional file 1: Figure S3). These initial sub-groups only need to accurately align cysteines. In the absence of any sequence similarity to a known structure, homologous cysteines cannot be reliably identified, although a number of prediction programs exist (Fariselli and Casadio 2001; Vullo and Frasconi 2004; Ceroni et al. 2006).

### Barcoding of cysteines with CysBar maintains the structural alignment of sequences

In order to merge these sub-group alignments, the non-homologous cysteines need to be distinguished from one another by retaining structural information in the sequences. To do this, alignment columns known to contain structurally homologous residues are replaced with 10 amino acid barcodes (Additional file 1: Figure S4a). This is done with either the user-friendly CysBar online web-tool or running the barcoder tool (*cysbar.py*) locally.

Two user inputs are required for each sub-group: firstly an alignment fasta file, and secondly the alignment positions to be barcoded. Each barcode is composed of the four least common amino acids (Wilkins et al. 1999) to prevent accidental mis-alignment to non-barcode sequence (Table 1).

**Table 1:** First 8 default barcode sequences used to replace homologous cysteine columns

ID	Sequence
bc.001	WWYHWYYHMM
bc.002	WHWMMHYHYY
bc.003	WWHHMWMMYW
bc.004	WHYYMMWMWM
bc.005	HWWMYHHMHW
bc.006	HMHYYWHHYM
bc.007	MMYMWMWHHW
bc.008	MYYHHMYWYY

The default set of barcode sequences minimise the likelihood of misalignment with the input sequences or with each other, based on principles developed for multiplex sequencing (Bystrykh 2012; Faircloth and Glenn 2012). Each sequence consists of the 4 least common amino acids in high-complexity sequences (Shannon entropy ≥1.8). Chance identity to random sequence is extremely low, with P(match) ≈ 10^−10^ for an alignment of 1000 sequences of length 1000. Additionally, to minimise the probability that barcodes erroneously align with one another, each differs from the others by at least 4 substitutions or indels (Hamming^+^ distance ≥4), with the first 8 barcodes differing from any others by 7 changes. The default set of 949 barcodes is ordered such that the sequences with higher robustness are used first. Equally robust barcodes are then ranked from high to low complexity. A full list of 948 default sequences, their Shannon entropy, and Hamming^+^ distance is contained in supplementary file *default_barcode_info.csv*.

Finally, the barcodes to be used are checked automatically for matches to the input sequences and the user notified that the next suitable barcode has been used instead. Custom barcodes of any length or sequence can be entered if required. The output fasta file is compatible with any standard alignment program and the residues in loops between the barcoded columns are free to realign (Additional file 1: Figure S4b). The identities of residues that were replaced with barcodes are stored in the fasta sequence ID for later reinsertion into the final alignment.

The barcoded sequences can then be re-aligned with any standard alignment algorithm, which allows the inter-cysteine loops to optimally align with the cysteine columns constrained by the barcode sequences. In this example Clustal Omega was used to align the barcoded defensin sequences since it is both accurate and scales well to large alignments (Sievers et al. 2011). The simultaneous alignment of all inter-cysteine regions, rather than aligning each block in isolation, allows the entire sequence to inform optimal alignment.

**Fig. 3:**
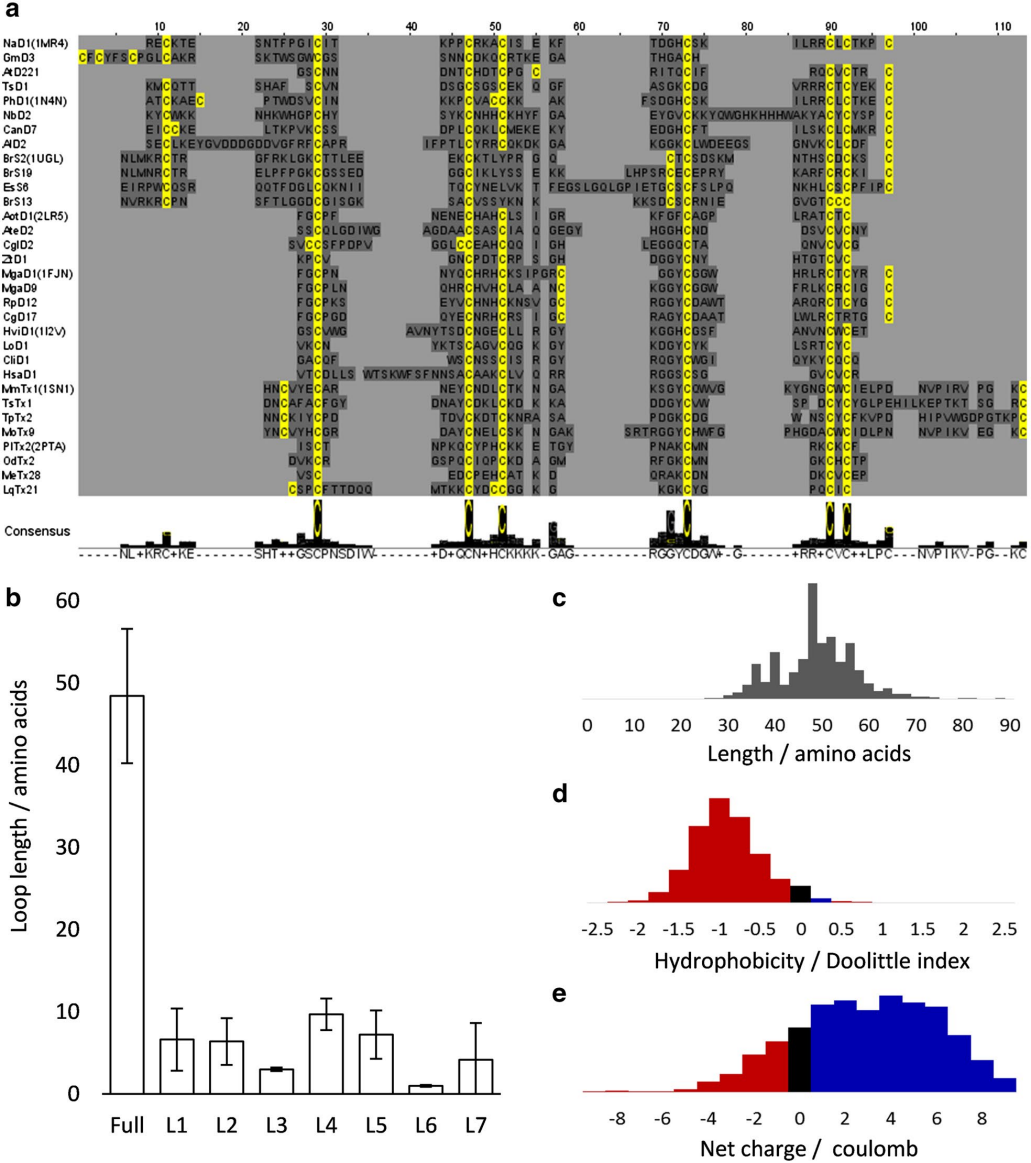
Final alignment. **a** Alignment of the sequences after barcodes have been replaced with original sequence columns by CysBar-r. Sequences *coloured* with cysteines in *yellow*, any other residue in *grey*, gaps in *light grey*. **b** Distribution of lengths of inter-cysteine loops. **c**–**e** Distribution of sequence length, hydrophobicity and net charge. Data from *oop_statistics.csv* processed by *loopproperties.xlsx*

Once the loops have been realigned, the second page of the CysBar web-tool or reconstructer function of the python tool will return the original columns in place of the barcodes to generate a final sequence alignment (Fig. 3). The only user input required for this is the realigned fasta file.

The final alignment by this method is superior to the initial, naïve alignments. Homologous cysteine alignment is retained at 100 %, rather than the 75–85 % by standard methods. Indels are also primarily predicted in loop regions, in agreement with the known evolutionary trends (Additional file 1: Figure S2). The sequence of the default barcodes do not affect the final alignment. When the method was repeated 10 times on the example dataset (using the first 80 default barcodes), no misalignment of barcodes to target sequences was observed. These alignments differed by <2 % (the same margin for repeating identical alignments with different random seeds). Repeating the method on a larger set of 965 sequences (Additional file 1: Figure S5), finds that the misalignment of cysteines and secondary structure by standard algorithms is slightly poorer than for the smaller example set (Additional file 1: Figure S6).

In addition to the final alignment, a.csv file is produced containing the biophysical properties for each loop of each sequence: length, hydrophobicity and net charge (Kyte and Doolittle 1982). These variables can also be user-defined if an alternative set of values is required. This file can be pasted into *loopproperties.xlsx* spreadsheet to display and graphically summarise the loop property results in Microsoft Excel (Additional file 1: Figure S7). This allows trends and similarities to be identified in the properties of sequences that are too diverse for easy comparison.

## Conclusion

Using the CysBar webtool and offline tools allows the alignment of previously unalignable protein sequences. This enables more robust bioinformatics on divergent, cysteine-rich sequences that previously had to be aligned manually. Most importantly, cysteine misalignment is abolished. A secondary effect of the improved cysteine alignment is that the inter-cysteine loop alignment is not disturbed by non-homologous loops. Finally, minimising manual alignment reduces user bias. Although extreme sequence diversity means that there is never enough information encoded in sequence or structure for perfect alignments, this method represents a significant improvement on previous protocols.

The CysBar web-tool provides a simple graphical user interface for performing the barcoding and reconstructing steps of the method. The biophysical properties are summarised by the *loopproperties.xlsx* spreadsheet interface and can be used to categorise sequences by their general properties even when their sequences are highly diverse. The annotated python script *cysbar.py* is also included in the supplementary materials along with a detailed readme containing step-by-step instructions and example data sets.

Defensin sequences have been used here as examples, however this method is equally applicable to other CRP superfamilies for which protein structures are available. Finally, the method is also applicable to sequences containing residues for which homology can be unambiguously assigned based on structural alignment (for example key catalytic residues).

## Availability and requirements

Project name: CysBar.

Project home page: http://CysBar.science.latrobe.edu.au/.

Scripts repository: http://github.com/TS404/CysBar/.

Operating system(s): The web-tool can be accessed through any web browsers. The python script runs on any Linux-like platform, such as the terminal on Linux/Unix/MacOS, or runs on Microsoft Windows with Python installed. The excel spreadsheet requires Microsoft Excel 2007 or newer.

Programming language: Python and PHP.

License: Academic Free License 3.0.

Any restrictions to use by non-academics: None beyond the general restriction against redistribution in the license.

## Additional files

10.1186/s40064-015-1609-z Misalignment by standard algorithms. **Figure S2.** Misalignment quantification. **Figure S3.** Alignments of sub-groups to closest sequence of known structure. **Figure S4.** Barcoded sequences. **Figure S5.** Misalignment by standard algorithms (larger data set). **Figure S6.** Misalignment quantification (larger data set). **Figure S7.** Summary of biophysical attributes by *loopproperties.xls*.
