# Supplementary material for: Structural homology guided alignment of cysteine rich proteins
Source: Springerplus. 2016 Jan 12;5:27. doi: 10.1186/s40064-015-1609-z (PMC4709342; doi:10.1186/s40064-015-1609-z)
Supplement: Supplementary file 1 — 10.1186/s40064-015-1609-z Misalignment by standard algorithms. Figure S2. Misalignment quantification. Figure S3. Alignments of sub-groups to closest sequence of known structure. Figure S4. Barcoded sequences. Figure S5. Misalignment by standard algorithms (larger data set). Figure S6. Misalignment quantification (larger data set). Figure S7. Summary of biophysical attributes by loopproperties.xls. [file 40064_2015_1609_MOESM1_ESM.pdf]

Supplementary information

Structural homology guided alignment of cysteine rich proteins

Thomas M A Shafee[1], Andrew J Robinson[2,3], Nicole van der Weerden[1], Marilyn A Anderson[1]

[1] Department of Biochemistry, La Trobe Institute of Molecular Sciences, La Trobe University, Melbourne, Australia, 3086
[2] College of Science, Health and Engineering, La Trobe University, Melbourne, Australia, 3086
[3] Life Sciences Computation Centre, Victorian Life Sciences Computation Initiative, Melbourne, Australia, 3053

Email: T.Shafee@LaTrobe.edu.au

SUPPLEMENTARY FIGURES

Figure S1 | Misalignment by standard algorithms ____ 2
Figure S2 | Misalignment quantification ____ 3
Figure S3 | Alignments of sub-groups to closest sequence of known structure ____ 4
Figure S4 | Barcoded sequences ____ 5
Figure S5 | Misalignment by standard algorithms (larger data set) ____ 6
Figure S6 | Misalignment quantification (larger data set) ____ 7
Figure S7 | Summary of biophysical attributes by *loopproperties.xls* ____ 8

SUPPLEMENTARY DATA FOLDERS:

1. Scripts, barcodes and readme
2. Failed initial alignments
3. DALI structural alignment
4. Aligned sub-groups
5. Barcoded sub-groups
6. Final combined alignment

Webtool implementation available at CysBar.science.latrobe.edu.au

Python scripts, readme, and example datasets available at Github.com/TS404/CysBar

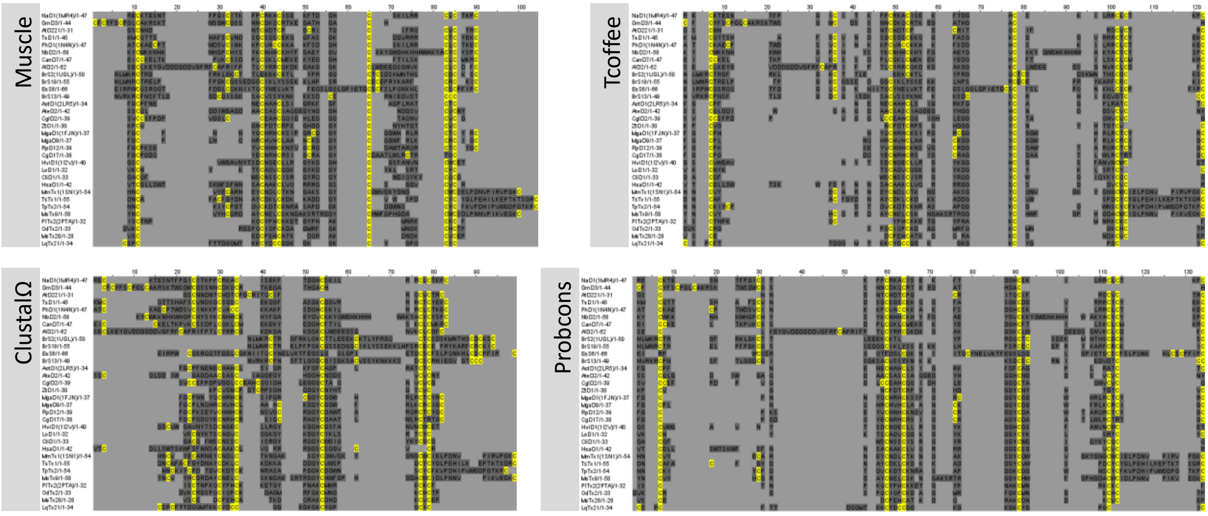

Figure S1 | Misalignment by standard algorithms

Alignments of structurally related defensins from plants, fungi and invertebrates using Muscle, Tcoffee, Clustal Ω or Probcons. Default settings on these algorithms generate conflicting, irreproducible alignments in which structurally homologous cysteines fail to align. Sequences coloured by JalView with cysteines in yellow, any other residue in grey, gaps in light grey. *Genbank accession numbers: 159162710, 571550504, 74820403, 332196243, 38492523, 6552502, 297824339, 158853052, 7209504, 557088590, 270381566, 392935432, 156179583, 88178907, 398388411, 193806528, 560135893, 555699991, 193806210, 159162452, 56462336, 49182286, 386642833, 51317001, 378748964, 399762321, 346655549, 6573542, 1173404, 487523606, 41017872.*

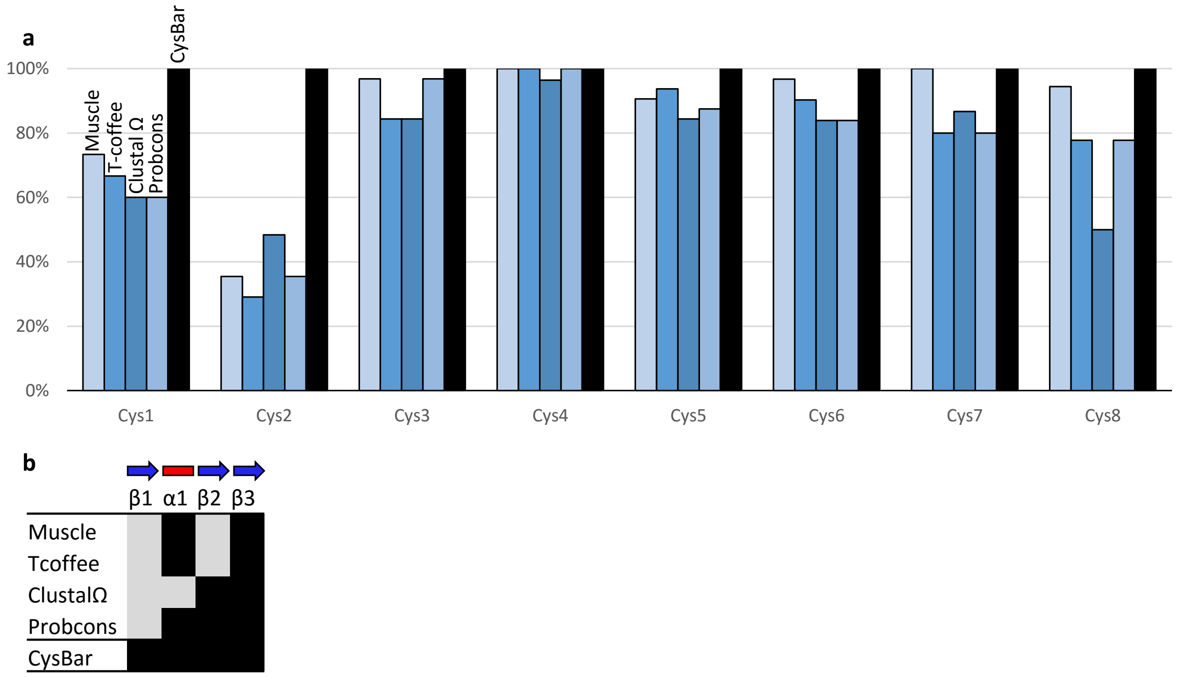

Figure S2 | Misalignment quantification

(a) Percentage of correct alignment for the 8 cysteines with known structural homology for each of Muscle, Tcoffee, Clustal Ω, Probcons and CysBar alignments. (b) For each alignment, secondary structural elements with one or more columns displaying >50% insertion or deletions in each alignment are indicated in light grey. (For alignments, see fig S1).

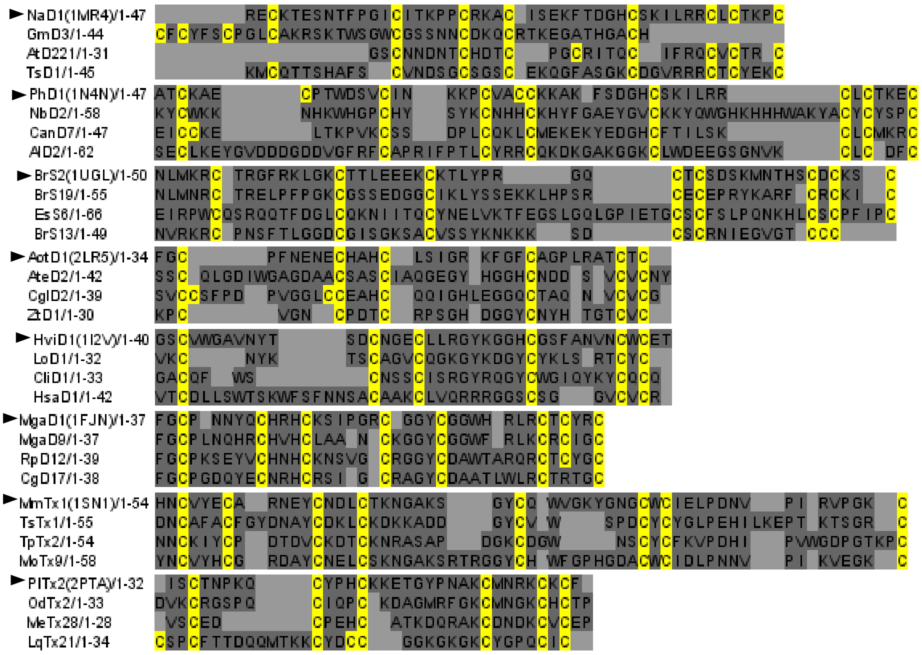

Figure S3 | Alignments of sub-groups to closest sequence of known structure

Alignments of sequences of unknown structure to their closest sequence of known structure (indicated by a black triangle) allows homologous cysteines to be found within sub-groups. Aligned with Muscle using default settings. Sequences coloured by JalView with cysteines in yellow, any other residue in grey, and gaps in light grey. *PDB accession numbers: 1MR4, 1N4N, 1UGL, 2LR5, 1I2V, 1FJN, 1SN1, 2PTA.*

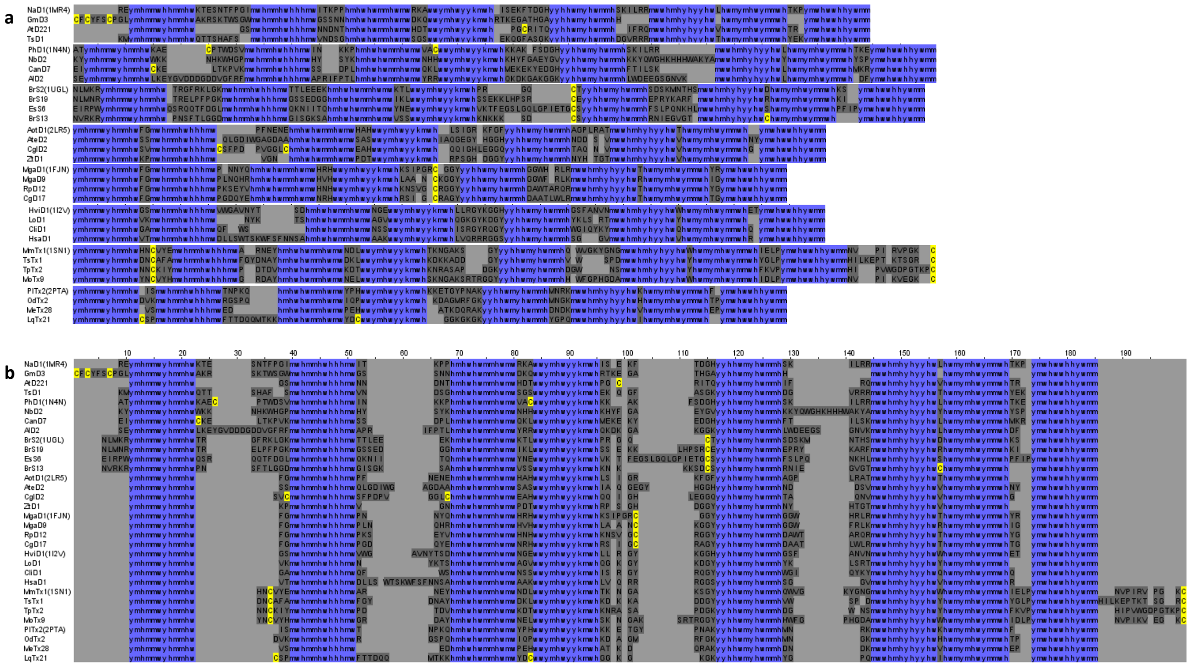

Figure S4 | Barcoded sequences

(**a**) Aligned sequence sub-groups with 8 columns barcoded by the CysBar web-tool or *barcoder.sh*. If the first cysteine was absent, *C1=0*, if the final cysteine was absent *C8=length+1*, if internal cysteines were absent, the homologous residue was chosen based on the structural alignment from Figure 2. (**b**) Full alignment after barcoded sub-groups were combined and re-aligned by Clustal Omega. Sequences coloured by JalView with cysteines in yellow, barcodes in blue, any other residue in grey, gaps in light grey.

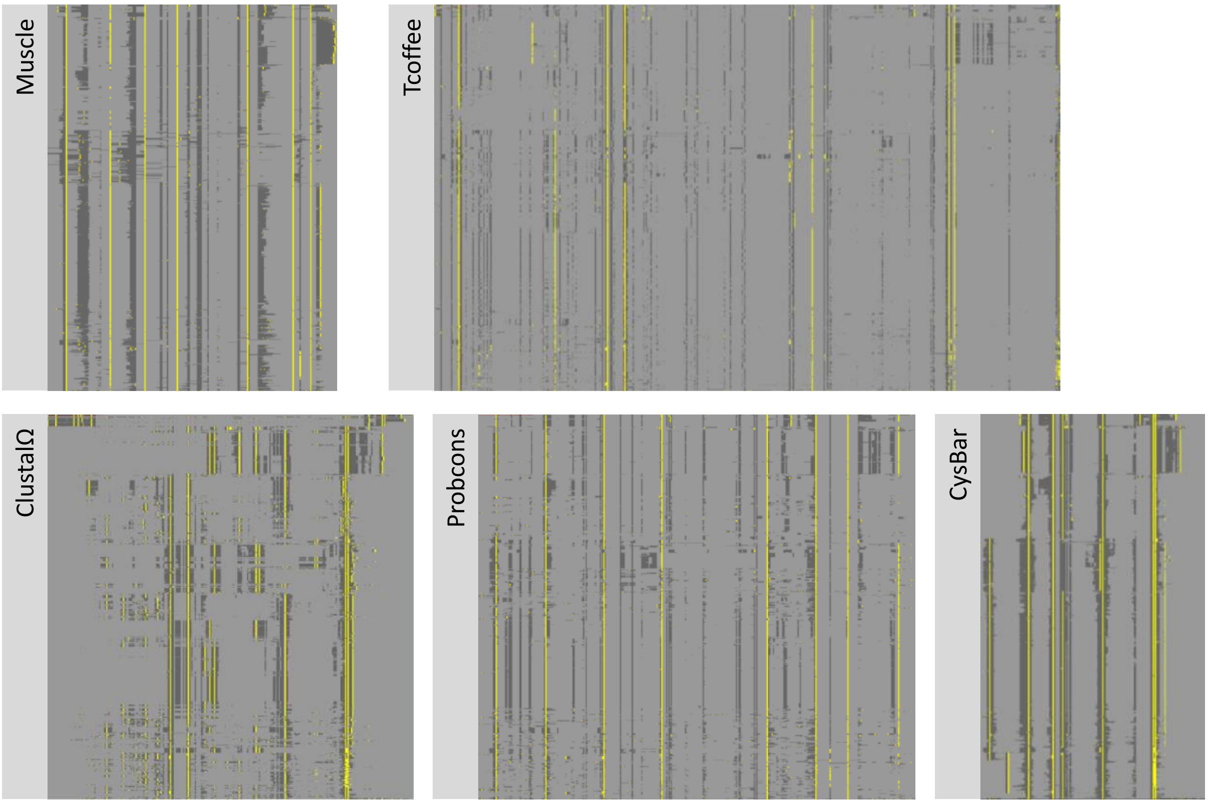

Figure S5 | Misalignment by standard algorithms (larger data set)
For larger alignment of 965 sequences, using default settings on Muscle, Tcoffee, Clustal Ω or Probcons generates conflicting, irreproducible alignments in which structurally homologous cysteines fail to align. The frequent mis-alignment of cysteines causes erroneous insertion and deletion predictions, leading to alignments with a large number of columns compared to the final CysBar alignment of the same sequences. Sequences coloured with cysteines in yellow, any other residue in grey, gaps in light grey.

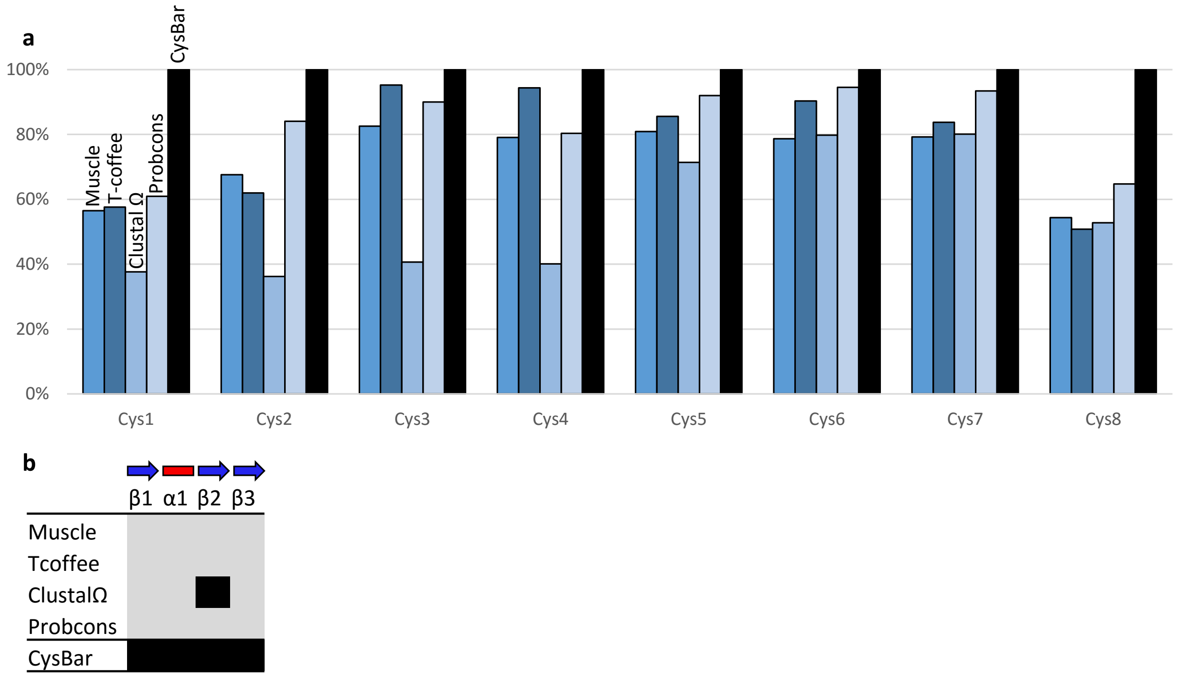

Figure S6 | Misalignment quantification (larger data set)
(a) Percentage of correct alignment for the 8 cysteines with known structural homology for each of Muscle, Tcoffee, Clustal Ω, Probcons and CysBar alignments. (b) For each alignment, secondary structural elements with one or more columns displaying >50% insertion or deletions in each alignment are indicated in light grey. (For alignments, see fig S5).

a

	A	B	C	D	E	F	G	H	I	J	K	L	M	N	O	P	Q	R	S	T	U	V	W	X	Y	Z	AA	AB	AC	AD	AE
1		full			N-ter			loop1			loop2			loop3			loop4			loop5			loop6			loop7			C-ter		
2	NAME	length	hydropat	charge	length	hydropat	charge	length	hydropat	charge	length	hydropat	charge	length	hydropat	charge	length	hydropat	charge	length	hydropat	charge	length	hydropat	charge	length	hydropat	charge	length	hydropat	charge
3	NaD1(1M	47	-1.082	6	2	-4	0	10	-0.78	0	5	-0.66	1	3	-2.2	2	9	-0.967	-1	6	-0.9	3	1	3.8	0	3	-2.067	1	0	1E-100	1E-100
4	GmD3/1-	44	-1.292	4	10	0.7571	0	10	-1.5	3	5	-1.8	0	3	-3.633	0	10	-1.37	1	1	-3.2	0	0	1E-100	1E-100	0	1E-100	1E-100	0	1E-100	1E-100
5	AtD221/	31	-1.357	1	0	1E-100	1E-100	2	-0.6	0	5	-2.94	-1	3	-2.467	-1	7	-1.033	1	4	-0.175	1	1	4.2	0	2	-2.6	1	0	1E-100	1E-100
6	TsD1/1-4	45	-1.284	3	2	-1	1	8	-0.638	0	5	-0.8	-1	3	-0.667	0	9	-1.311	1	6	-2.2	2	1	-0.7	0	3	-2.9	0	0	1E-100	1E-100
7	PhD1(1N	47	-0.943	6	2	0.55	0	10	-0.989	-1	5	-1.68	2	3	3	0	9	-1.667	2	6	-0.9	3	1	3.8	0	3	-2.7	0	0	1E-100	1E-100
8	NbD2/1-	58	-1.886	9	2	-2.6	1	10	-2.54	3	5	-2.1	1	3	-3.3	0	10	-0.52	0	16	-2.069	4	1	-1.3	0	3	-1.233	0	0	1E-100	1E-100
9	CanD7/1	47	-0.968	2	2	0.5	-1	9	-1.188	2	5	-0.58	-1	3	-1.2	1	10	-2.48	-2	6	0.95	1	1	3.8	0	3	-2.167	2	0	1E-100	1E-100
10	AlD2/1-6	62	-1.028	-2	2	-2.15	-1	17	-0.829	-4	8	0.5625	1	3	-3.433	2	10	-2.2	3	11	-1.127	-2	1	3.8	0	2	-0.35	-1	0	1E-100	1E-100
11	BrS2(1U	50	-1.576	6	5	-1.24	2	9	-1.3	4	7	-1.714	-2	3	-0.267	1	6	-2.14	1	9	-1.7	0	1	-3.5	-1	2	-2.35	1	0	1E-100	1E-100
12	BrS19/1-	55	-1.349	6	5	-1.16	1	9	-1.067	1	7	-1.4	-2	3	1.4667	1	12	-2.064	1	8	-1.838	2	1	-4.5	1	2	0.3	1	0	1E-100	1E-100
13	EsS6/1-6	66	-0.598	0	5	-1.2	0	10	-1.38	0	7	-0.871	1	3	-2.767	-1	19	-0.083	-1	9	-0.678	1	1	-0.8	0	4	1.025	0	0	1E-100	1E-100
14	BrS13/1-	49	-1.066	6	5	-2.44	3	9	-0.478	-1	7	0	1	3	0.8667	0	9	-3.025	3	8	-0.538	0	1	1E-100	0	0	1E-100	1E-100	0	1E-100	1E-100
15	AotD1(2	34	-0.414	1	0	1E-100	1E-100	2	1.2	0	6	-2.133	-2	3	-1.533	0	9	0.4333	2	7	0.0286	1	1	-0.7	0	0	1E-100	1E-100	0	1E-100	1E-100
16	AtcD2/1	42	-0.5	-5	0	1E-100	1E-100	2	-0.8	0	12	0.0917	-2	3	0.0667	0	11	-0.909	-1	5	-1.42	-2	1	4.2	0	2	-2.4	0	0	1E-100	1E-100
17	CglD2/1	39	-0.226	-3	0	1E-100	1E-100	3	1.7	0	10	0.2778	-1	3	-1.633	-1	10	-1.01	-1	5	-0.34	0	1	4.2	0	1	-0.4	0	0	1E-100	1E-100
18	ZtD1/1-3	30	-1.363	0	0	1E-100	1E-100	2	-2.75	1	3	0.1	0	3	-1.933	-1	9	-1.789	0	6	-1.633	0	1	4.2	0	0	1E-100	1E-100	0	1E-100	1E-100
19	MgaD1(1	37	-1.631	6	0	1E-100	1E-100	2	1.2	0	5	-2.68	0	3	-3.633	1	10	-0.978	2	7	-1.443	2	1	-0.7	0	2	-2.9	1	0	1E-100	1E-100
20	MgaD9/	37	-0.631	5	0	1E-100	1E-100	2	1.2	0	6	-2.083	1	3	-0.733	0	9	-0.263	1	7	-0.5	2	1	-4.5	1	2	2.05	0	0	1E-100	1E-100
21	RpD12/1	39	-1.348	3	0	1E-100	1E-100	2	1.2	0	6	-1.15	0	3	-3.3	0	10	-1.222	2	8	-1.75	1	1	-0.7	0	2	-0.85	0	0	1E-100	1E-100
22	CgD17/1	38	-1.061	2	0	1E-100	1E-100	2	1.2	0	6	-2.3	-2	3	-3.733	1	9	-0.7	2	8	0.2	0	1	-0.7	0	2	-0.55	0	0	1E-100	1E-100
23	HviD1(1I2	40	-0.603	-1	0	1E-100	1E-100	2	-0.6	0	10	-0.09	-1	3	-2.467	-1	9	-0.722	2	7	0.0857	0	1	-0.9	0	2	-2.1	-1	0	1E-100	1E-100
24	LoD1/1-3	32	-1.215	5	0	1E-100	1E-100	2	0.15	1	5	-2.04	1	3	1.8667	0	9	-2.067	1	6	-1.233	2	1	-1.3	0	0	1E-100	1E-100	0	1E-100	1E-100
25	CliD1/1-3	33	-1.189	3	0	1E-100	1E-100	2	0.7	0	4	-0.6	0	3	-1.7	0	9	-1.356	2	7	-0.971	1	1	-3.5	0	1	-3.5	0	0	1E-100	1E-100
26	HsaD1/1-	42	-0.283	5	0	1E-100	1E-100	2	1.75	0	16	-0.319	0	3	-0.1	1	9	-1.178	3	4	0.65	0	1	4.2	0	1	-4.5	1	0	1E-100	1E-100
27	MmTx1(1	54	-1.054	1	0	1E-100	1E-100	6	-1.46	-1	5	-2.2	0	3	-1.067	-1	9	-1.456	2	9	-1.122	1	1	-0.9	0	4	0.8	-1	10	-0.289	2
28	TsTx1/1-	55	-1.138	-2	0	1E-100	1E-100	6	-0.12	-1	7	-0.771	-1	3	-1.2	0	9	-2.456	0	5	-0.52	-1	1	-1.3	0	4	0.125	0	13	-1.242	2
29	TpTx2/1	54	-1.324	0	0	1E-100	1E-100	6	-1.54	1	5	-1.02	-2	3	-2.7	0	10	-1.85	2	5	-1.82	-1	1	-1.3	0	4	0.375	1	13	-0.758	0
30	MoTx9/	58	-1.064	2	0	1E-100	1E-100	6	-1.02	0	5	-1.58	0	3	-1.067	-1	13	-1.792	4	9	-0.956	-1	1	-0.9	0	4	0.8	-1	10	-0.433	1
31	PlTx2(2F	32	-1.831	6	0	1E-100	1E-100	2	1.85	0	5	-2.64	1	3	-2.033	0	10	-2.09	2	4	-2.5	2	1	-3.9	1	1	2.8	0	0	1E-100	1E-100
32	OdTx2/1	33	-1.337	4	0	1E-100	1E-100	3	-1.067	0	5	-2.16	1	3	-0.2	0	9	-1.122	2	4	-1.475	1	1	-3.2	0	2	-1.15	0	0	1E-100	1E-100
33	McTx28	28	-1.982	-3	0	1E-100	1E-100	2	1.7	0	2	-3.5	-2	3	-2.767	-1	8	-2.05	2	4	-3.6	-1	1	4.2	0	2	-2.55	-1	0	1E-100	1E-100
34	LqTx21/1	34	-1.481	3	0	1E-100	1E-100	3	-1.2	0	10	-1.57	1	3	-2.4	-1	7	-1.9	3	4	-1.7	0	1	4.5	0	0	1E-100	1E-100	0	1E-100	1E-100

b

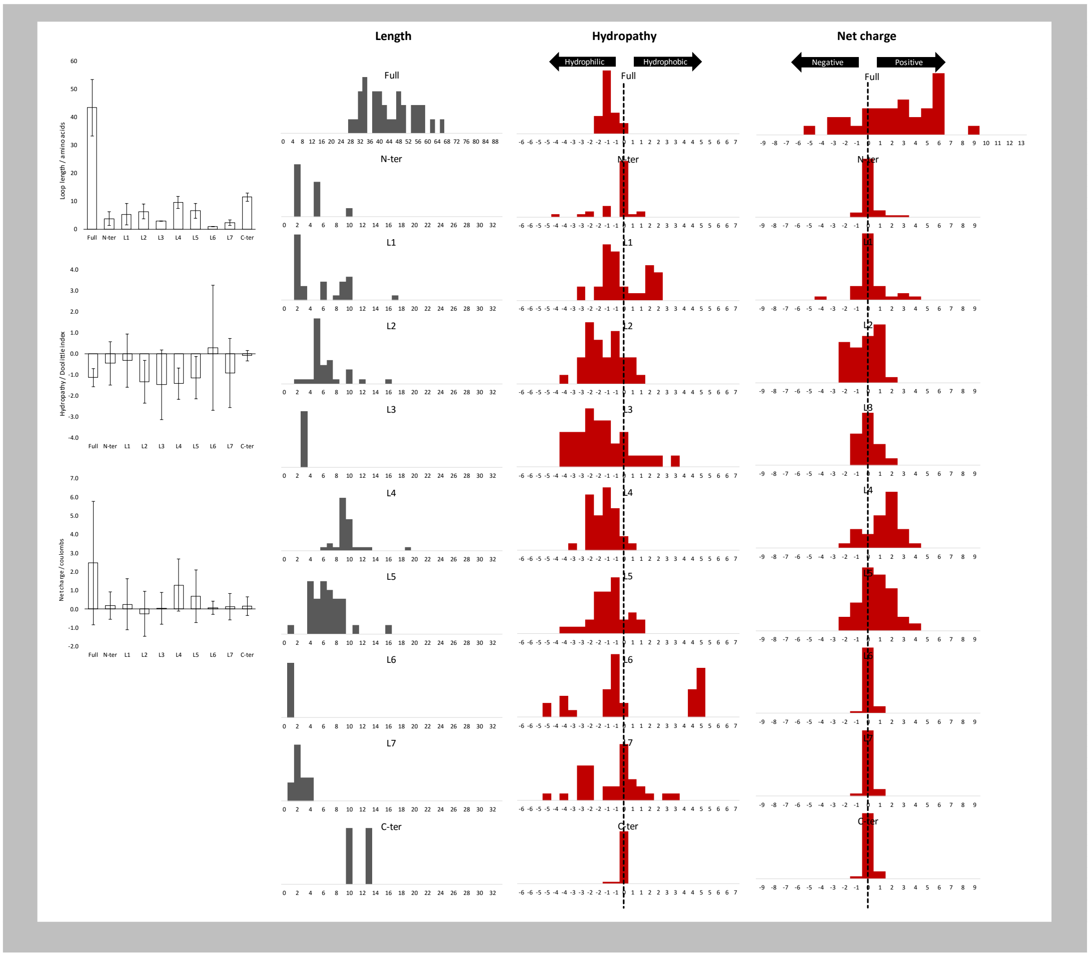

Figure S7 | Summary of biophysical attributes by *loopproperties.xls*

Screenshots of *loopproperties.xlsx* when opening the *loop_statistics.csv* data file. (**a**) Raw data in *loop_statistics.csv* file is for length, hydrophobicity and net charge of the full sequence and each inter-cysteine loop. Highlighted in pink are loops that contain no sequence so hydrophobicity or charge cannot be calculated. Cysteines are assumed to be involved in disulphide bonds. (**b**) Data is processed by *loopproperties.xlsx* into bar charts displaying average properties and a full set of histograms of length, hydrophobicity, and charge for each loop.
